# A Validated Nomogram That Predicts Prognosis of Autoimmune Encephalitis: A Multicenter Study in China

**DOI:** 10.3389/fneur.2021.612569

**Published:** 2021-04-08

**Authors:** Yueqian Sun, Guoping Ren, Jiechuan Ren, Wei Shan, Xiong Han, Yajun Lian, Tiancheng Wang, Qun Wang

**Affiliations:** ^1^Department of Neurology, Beijing Tiantan Hospital, Capital Medical University, Beijing, China; ^2^China National Clinical Research Center for Neurological Diseases, Beijing, China; ^3^Beijing Institute for Brain Disorders, Collaborative Innovation Center for Brain Disorders, Capital Medical University, Beijing, China; ^4^Department of Neurology, Henan Provincial People's Hospital, Henan, China; ^5^Department of Neurology, The First Affiliated Hospital of Zhengzhou University, Henan, China; ^6^Department of Neurology, Lanzhou University Second Hospital, Lanzhou, China; ^7^Beijing Key Laboratory of Neuromodulation, Beijing, China

**Keywords:** nomogram, autoimmune encephalitis, prognosis, modified Rankin Scale, prediction

## Abstract

The aim of this retrospective study was to derive and validate a reliable nomogram for predicting prognosis of autoimmune encephalitis (AE). A multi-center retrospective study was conducted in four hospitals in China, using a random split-sample method to allocate 173 patients into either a training (*n* = 126) or validation (*n* = 47) dataset. Demographic, radiographic and therapeutic presentation, combined with clinical features were collected. A modified Rankin Scale (mRS) at discharge was the principal outcome variable. A backward-stepwise approach based on the Akaike information criterion was used to test predictors and construct the final, parsimonious model. Multivariable analysis was conducted using logistic regression to develop a prognosis model and validate a nomogram using an independent dataset. The performance of the model was assessed using receiver operating characteristic curves and a Hosmer-Lemeshow test. The final nomogram model considered age, viral prodrome, consciousness impairment, memory dysfunction and autonomic dysfunction as predictors. Model validations displayed a good level of discrimination in the validation set: area under the Receiver operator characteristic curve = 0.72 (95% Confidence Interval: 0.56–0.88), Hosmer–Lemeshow analysis suggesting good calibration (chi-square: 10.33; *p* = 0.41). The proposed nomogram demonstrated considerable potential for clinical utility in prediction of prognosis in autoimmune encephalitis.

## Introduction

Autoimmune encephalitis (AE) comprises a heterogeneous group of acute or subacute encephalitic syndromes caused by an autoimmune etiology, rather than traditional infectious pathogenesis, characterized by prominent neuropsychiatric symptoms (1). Autoantibodies related to AE include those directed against N-methyl-D-aspartate receptor (NMDAR), leucine-rich glioma inactivated 1 (LGI1), γ-aminobutyric acid receptor-B (GABAbR), and contactin-associated protein like 2 (CASPR2), etc. (2). Typical clinical manifestations include seizures, psychiatric and behavioral disorders, disturbances in consciousness, movement abnormalities, autonomic disorders, and memory and cognitive deficits (3). While ~80% of patients with AE recover well following immunotherapy, there is still a percentage of patients that do not respond to these therapies, and the case mortality rate of subtype of AE is between 2.9 and 10% (4–6). Therefore, it becomes particularly important to pursue early evaluation of prognosis, which enables a more focused management of complications, including conditions causing serious illness that require intensive care (7). Understanding which factors influence prognosis is required to provide an informative perspective on personalized patient care. Patients with poor prognosis should receive early intervention (8, 9). In contrast, overtreatment in patients with good prognosis could lead to potential harm and more substantial medical costs. Several well-defined factors such as consciousness impairment have been demonstrated to influence response to immunotherapy in AE (10). A straightforward prognosis model based on clinical and biologic features of patients with AE enables potentially benefit to select appropriate treatment. Predicting positive immunotherapeutic response justifies expedited implementation of immunotherapy potentially improving neurologic dysfunction (11, 12). At present, several prognostic models (13–15) have been constructed to identify predictors associated with prognosis in AE. However, some of them are restricted to one single subtype, and some of them include a relatively high number of existing variables, which hinders the practical usage and further clinical application. Integration of various prognostic factors into a nomogram is considered a simple-to-implement visualization tool for implementation of individual predictions. However, till now, nomograms for the prognosis of AE have not been clearly characterized. Additional development is required to accurately predict individual risk of poor prognosis for AE based on a nomogram.

The present study aimed to develop and validate a prognostic nomogram for AE patients using an independent set of patient data. We hypothesized that factors identified as predictors of poor prognosis could be discretely visualized using a nomogram.

## Materials and Methods

### Study Design and Participants

The present investigation was a multicenter retrospective study of AE patients. Medical records of patients were collected from Beijing Tiantan Hospital, Henan Provincial People's Hospital, the First Affiliated Hospital of Zhengzhou University and Lanzhou University Second Hospital. The study was reviewed and approved by the local ethics committee within each center.

### Data Collection

Inclusion criterion: (1) patients with an initial diagnosis of AE who accepted inpatient treatment from May 2012 to May 2018. The basis of diagnosis was based on the clinical diagnostic criteria for AE, as suggested by Mittal and Graus in 2016 (16); (2) received first-line immunotherapy during the inpatient stay, including corticosteroids, intravenous immunoglobulins (IVIG), or plasma exchange. The following exclusion criteria was implemented: (1) negative antibody testing both in serum and CSF; (2) having another autoimmune disease; (3) incomplete clinical data; (4) modified Rankin Scale (mRS) > 0 before the onset of disease.

Neurological outcomes were evaluated using mRS (17). According to mRS score at discharge, patients were divided onto one of two groups: patients with an mRS ≤ 2, defined as “good prognosis,” representing a continuum of function without disability (mRS 0) to slight disability but able to live independently (mRS 2). Conversely, “poor prognosis” (defined as mRS > 2) spanned a range from moderate disability requiring help for entirely independent living but able to walk independently (mRS 3), to severe disability, being bed bound and fully dependent on continuous nursing care (mRS 5) and death (mRS 6).

The association between the following factors and functional status at discharge was analyzed: (1) demographics (sex and age); (2) clinical AE signs (viral prodrome including fever, headache, respiratory tract infection and infection of digestive canal, consciousness impairment, behavioral changes, memory dysfunction, speech disorders, sleep dysfunction, seizures, autonomic dysfunction and movement disorders); (3) laboratory and radiographic findings (CSF protein, white blood cell (WBC) count and abnormal cranial magnetic resonance imaging (MRI) findings); (4) other clinical features [diagnosis of tumor, immunotherapy latency (the time interval from onset to the initiation of immunotherapy)]. MRI scans were classified as abnormal based on T2 or fluid-attenuated inversion recovery (FLAIR) hyperintensity in one or both medial temporal lobes, multiple inflammation or demyelination involving gray and white matter (16).

### Sample Size

In prediction studies, sample size was strongly dependent on the number of outcome events. According to a number of empirical investigations (18), at least 10 outcome events per variable (EPV) were acquired. In the present study, sample size and the numbers of events approximately followed the minimum required and thus provided sufficient power.

### Statistical Analysis

All data were analyzed statistically in R version 3.6.1 and STATA version 15.0. The caret R package (19) was used to extract training and validation datasets from samples identified as positive based on a probability of 3:1 and seed set to 123 to ensure consistency of results. The training set was used for model building, while the test set was used for evaluation. Univariate binary logistic regression models were performed to assess independent predictors of poor prognosis in AE. Continuous data was summarized using median (quartiles), and categorical data as numbers (percentages). Chi-square analysis or Fisher exact test was used for univariate analysis. Those factors that had a *P*-value < 0.3 were included in multivariable logistic regression analysis, and the training dataset was used to develop the prognostic models for the multivariable logistic regression. Nonsignificant variables were eliminated in a stepwise fashion. The final model minimized the score with an Akaike information criterion (AIC) (20) having fewest variables. The nomogram was derived from the results of multivariate logistic analysis using R.

In the nomogram, the regression coefficients of all independent variables were used to determine the proportion of scores, and a score level was assigned for each independent variable. For each patient, the nomogram helped estimate the total score and then predict the probability of poor prognosis. Put it another way, the vertical projection of the points on the axis corresponds to a single score. Then, sum the points received for each variable, and locate the number on the “Total Points” axis. Each patient is given a score, the higher the score the poorer the prognosis. The nomogram created based on the training dataset was used for calculation of outcome probability in the validation dataset. The prediction model was subsequently assessed based on nomogram discrimination and calibration using the validation set. Discrimination was assessed by partial area under the receiver-operator characteristic (ROC) curve and calibration was ensured through a Hosmer–Lemeshow (HL) goodness-of-fit test. Data were presented as adjusted odds ratios (ORs) with 95% confidence intervals (CIs). A two-sided *p*-value of <0.05 was deemed significant.

## Results

**Figure 1:**
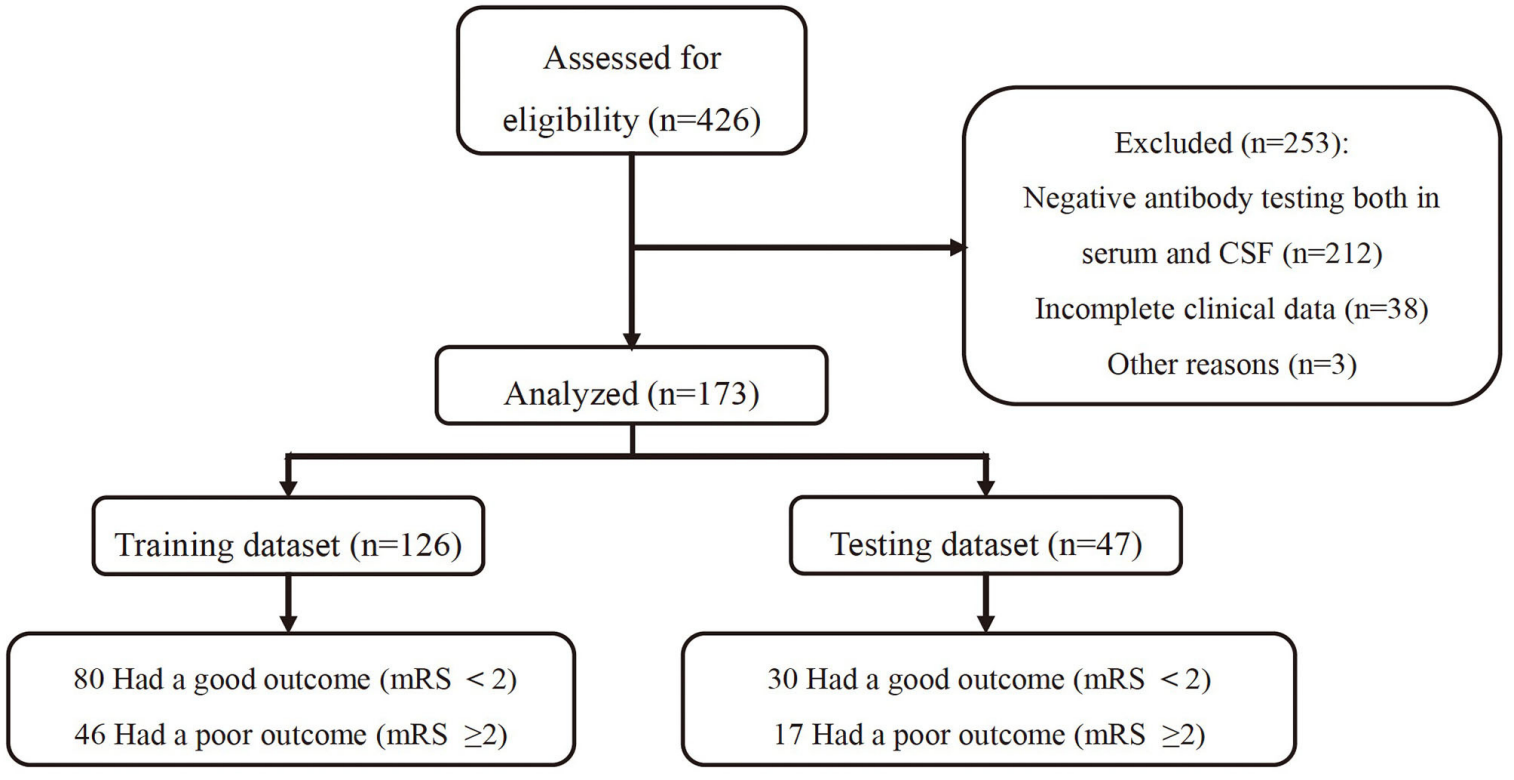
Numbers of participants enrolled and outcomes in the training and validation data sets.

**Figure 2:**
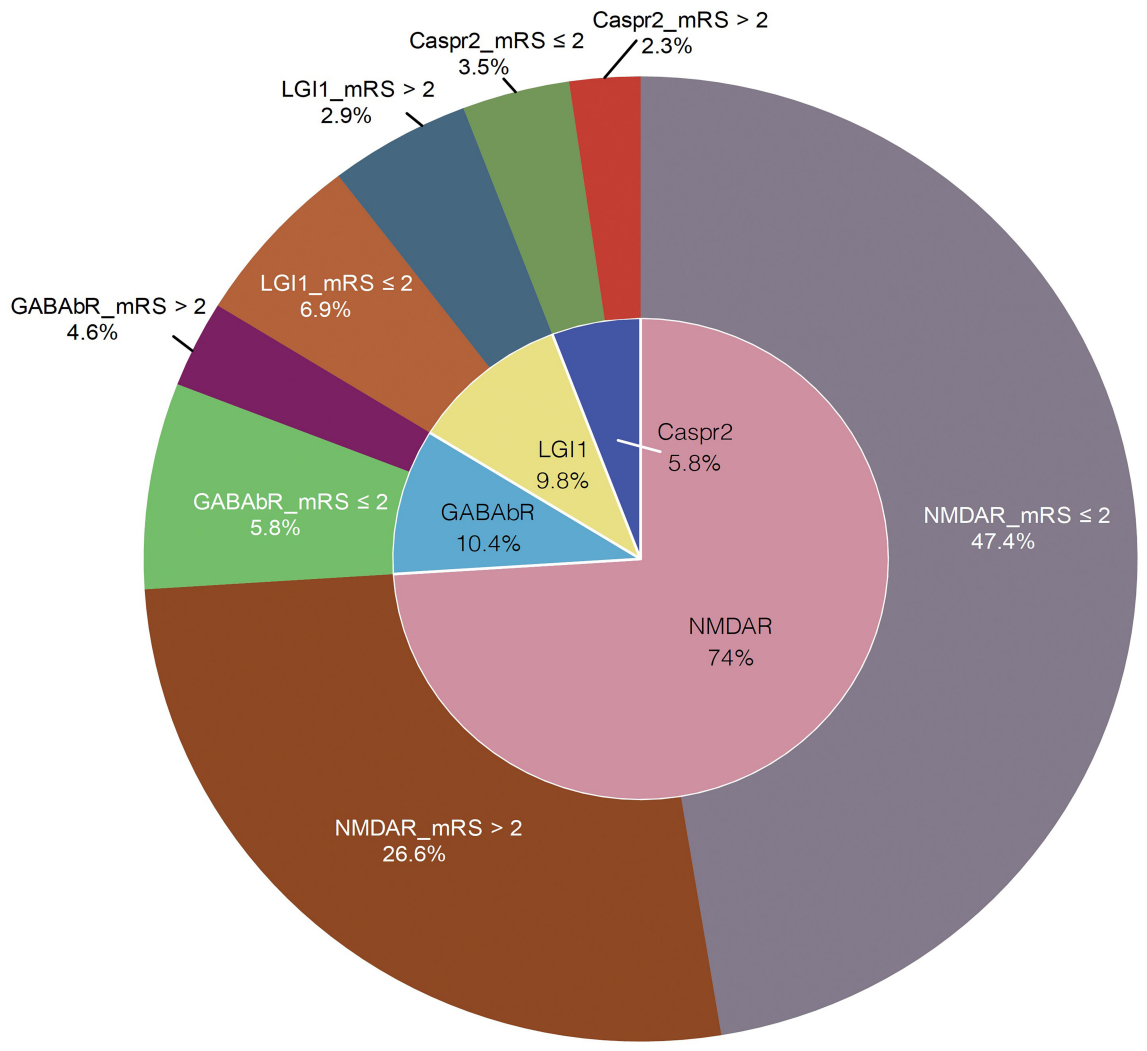
Percentage of prognosis of subtype of AE.

A total of 426 patients fulfilling all eligibility criteria (Figure 1) were assessed. After exclusion of ineligible patients, 173 subjects were included in the final analysis, including 92 men (53.2%) and 81 women (46.8%), with a median age of 36.3 years (range: 2.5–84years). As we have shown in Figure 2, the inner ring around the pie chart showed the distribution of the subtype of AE, and the outer ring depicted the percentage distribution of good and poor outcome in each category (Figure 2). Obviously, NMDAR encephalitis had the largest proportion of all AE patients (74%) and all four categories the proportion of patients with favorable outcome was larger than those with unfavorable outcome. Of the 173 patients, viral prodromal symptoms were found in 53.8% individuals, consisting of headache, fever, upper respiratory tract symptoms or diarrhea. Twenty-four percent of patients had depressed levels of consciousness. Autonomic dysfunction was observed in 17.3%. Memory dysfunction accounted for 41.6% of cases. At discharge, 63.6% of patients had a mRS score > 2.

Finally, all of the patients were randomly assigned to the training (*n* = 126) or validation cohort (*n* = 47). Data from training cohort were used to build the nomogram, and validation cohort to assess model performance. The proportion of patients with poor prognosis in the training and validation datasets was 36.5 and 36.2%, respectively. Randomization was successful with no statistically significant differences in any parameters of the two datasets (Table 1). In the training data set, the median age was 36.7 years (range: 2.5–84 years), 42.1% had cognitive impairment, 55.6% had vital prodrome, 24.6% had consciousness impairment and 19.0% had autonomic dysfunction.

**Table 1:** Summary of study variables stratified by data set.

	**Dataset**		
**Variable**	**Training**	**Testing**	***P*-value**
Sample size	126 (72.8)	47 (27.2)	NA
Male gender	68 (54.0)	24 (51.1)	0.74
Age, yr	36.7 ± 18.6	35.3 ± 19.4	0.68
Antibodies			0.91
NMDAR	91 (72.2)	37 (78.7)	
LGI1	13 (10.3)	4 (8.5)	
GABAbR	14 (11.1)	4 (8.5)	
CASPR2	8 (6.3)	2 (4.3)	
Viral prodrome	70 (55.6)	23 (48.9)	0.49
Memory dysfunction	53 (42.1)	19 (40.4)	0.86
Speech disorders	31 (24.6)	11 (23.4)	0.87
Behavioral changes	79 (62.7)	31 (66.0)	0.73
Seizures	78 (61.9)	30 (63.8)	0.86
Consciousness impairment	31 (24.6)	11 (23.4)	0.87
Sleep dysfunction	22 (17.5)	13 (27.7)	0.14
Movement disorder	42 (33.3)	11 (23.4)	0.27
Autonomic dysfunction	24 (19.0)	6 (12.8)	0.38
CSF WBC count (>20 cell/μL)	50 (39.7)	18 (38.3)	0.87
CSF protein (>30 mg/dL)	65 (51.6)	24 (51.1)	0.95
Abnormal MRI	87 (69.0)	34 (72.3)	0.71
Tumor	9 (7.1)	5 (10.6)	0.53
Immunotherapy latency (>4 wk)	61 (48.4)	21 (44.7)	0.73
Outcome (mRS >2)	46 (36.5)	17 (36.2)	0.97

*Data presented as n (%) or mean ± standard deviation*.

*NA, not applicable; NMDAR, N-methyl-D-aspartate receptor; LGI1, leucine-rich glioma inactivated 1; GABAbR, γ-aminobutyric acid receptor-B; CASPR2, contactin-associated protein like 2; CSF, cerebrospinal fluid; WBC, white blood cell; MRI, magnetic resonance imaging; mRS, modified Rankin Scale*.

*Continuous variables were compared using Mann Whitney U test and categorical variables were compared using Fisher's exact test*.

**Figure 3:**
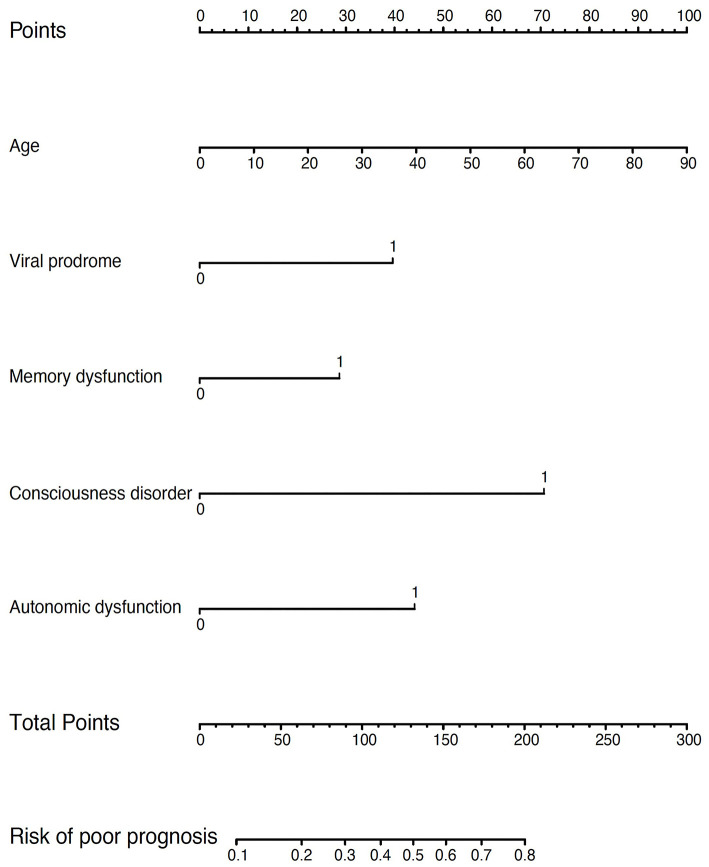
Nomogram to estimate the probability of poor prognosis in AE. A nomogram for poor prognosis prediction was developed and integrated with the predictors. Find the predictor points on the upper most point score that correspond to each patient variable and sum them. The total points projected to the bottom score indicate the percentage of probability of poor prognosis. For example, a 20-year-old male patient with memory dysfunction, consciousness impairment, autonomic dysfunction and no viral prodrome would have a score of 162.5 (22.5 + 27.5 + 70 + 42.5), corresponding to an 65% probability of poor prognosis.

Table 2 indicates associations between each predictor and poor prognosis for the training dataset. All analyzed factors demonstrated a *P* < 0.3 in univariate analysis were included in the multivariate analysis. As shown in Table 3, after variable selection, only five were identified in the final multivariable prediction model, including age, viral prodrome, memory dysfunction, consciousness impairment, and autonomic dysfunction. As mentioned in method section, our model has five predictor variables, so the number of events would be a minimum sample of 50. The actual number of outcome events in present study was 46, which was within the acceptable range. Consciousness impairment was found to be a significant independent predictor of poor prognosis (*p* = 0.003). Hence, a model incorporating these five characteristics was developed and visualized as a nomogram (Figure 3); based on the nomogram, we first scored each variable based on the top scale of the nomogram and then summed the points of each factor. The total score ranging from 0 to 300 was used to predict the risk of poor prognosis. The predicted probability was estimated for each object and ranged from 10 to 80%.

**Table 2:** Results of univariate binary logistic regression analysis of study variables vs. mRS for training dataset.

	**mRS score**		
**Variable**	**≤2 (*n* = 80)**	**>2 (*n* = 46)**	***P*-value**
Male gender	46 (57%)	22 (48%)	0.35
Age, yr	33.73 (17.78)	41.74 (19.07)	0.02
Antibodies			0.98
NMDAR	57 (71%)	34 (74%)	
LGI1	9 (11%)	4 (9%)	
GABAbR	9 (11%)	5 (11%)	
CASPR2	5 (6%)	3 (7%)	
Viral prodrome	41 (51%)	29 (63%)	0.26
Memory dysfunction	30 (38%)	23 (50%)	0.19
Speech disorders	20 (25%)	11 (24%)	0.89
Behavioral changes	46 (57%)	33 (72%)	0.13
Seizures	50 (63%)	28 (61%)	0.85
Consciousness impairment	12 (15%)	19 (41%)	<0.01
Sleep dysfunction	12 (15%)	10 (22%)	0.34
Movement disorder	24 (30%)	18 (39%)	0.33
Autonomic dysfunction	9 (11%)	15 (33%)	<0.01
CSF WBC count (>20 cell/μL)	27 (34%)	23 (50%)	0.09
CSF protein (>30 mg/dL)	39 (49%)	26 (57%)	0.46
Abnormal MRI	51 (64%)	36 (78%)	0.11
Tumor	4 (5%)	5 (11%)	0.29
Immunotherapy latency (>4 wk)	36 (45%)	25 (54%)	0.36

*Data presented as n (%) or mean ± standard deviation*.

*NA, not applicable; mRS, modified Rankin Scale; NMDAR, N-methyl-D-aspartate receptor; LGI1, leucine-rich glioma inactivated 1; GABAbR, γ-aminobutyric acid receptor-B; CASPR2, contactin-associated protein like 2; CSF, cerebrospinal fluid; WBC, white blood cell; MRI, magnetic resonance imaging*.

*Univariate binary logistic regression analysis was conducted using either the Fisher exact test or Mann-Whitney U-test; the P-value was derived from bivariate association analyses between each study variables and the mRS score*.

**Table 3:** Predictors for poor prognosis in final regression model for training data set.

**Intercept and variable**	**β Coefficient**	**OR (95% CI)**	***P*-value**
Intercept	−2.653	NA	NA
memory dysfunction	0.578	1.782 (0.754–4.214)	0.188
Age, mean (SD), yr	0.022	1.023 (0.999–1.047)	0.065
Consciousness impairment	1.425	4.157 (1.623–10.645)	0.003
Autonomic dysfunction	0.889	2.433 (0.872–6.792)	0.09
Viral prodrome	0.799	2.223 (0.941–5.253)	0.069
**Area under ROC curve**			
Training data set	0.76 (0.67–0.84)		
Validation data set	0.72 (0.56–0.88)		

*AE, autoimmune encephalitis; CI, confidence interval; NA, not applicable; OR, odds ratio; ROC, receiver operating characteristic*.

**Figure 4:**
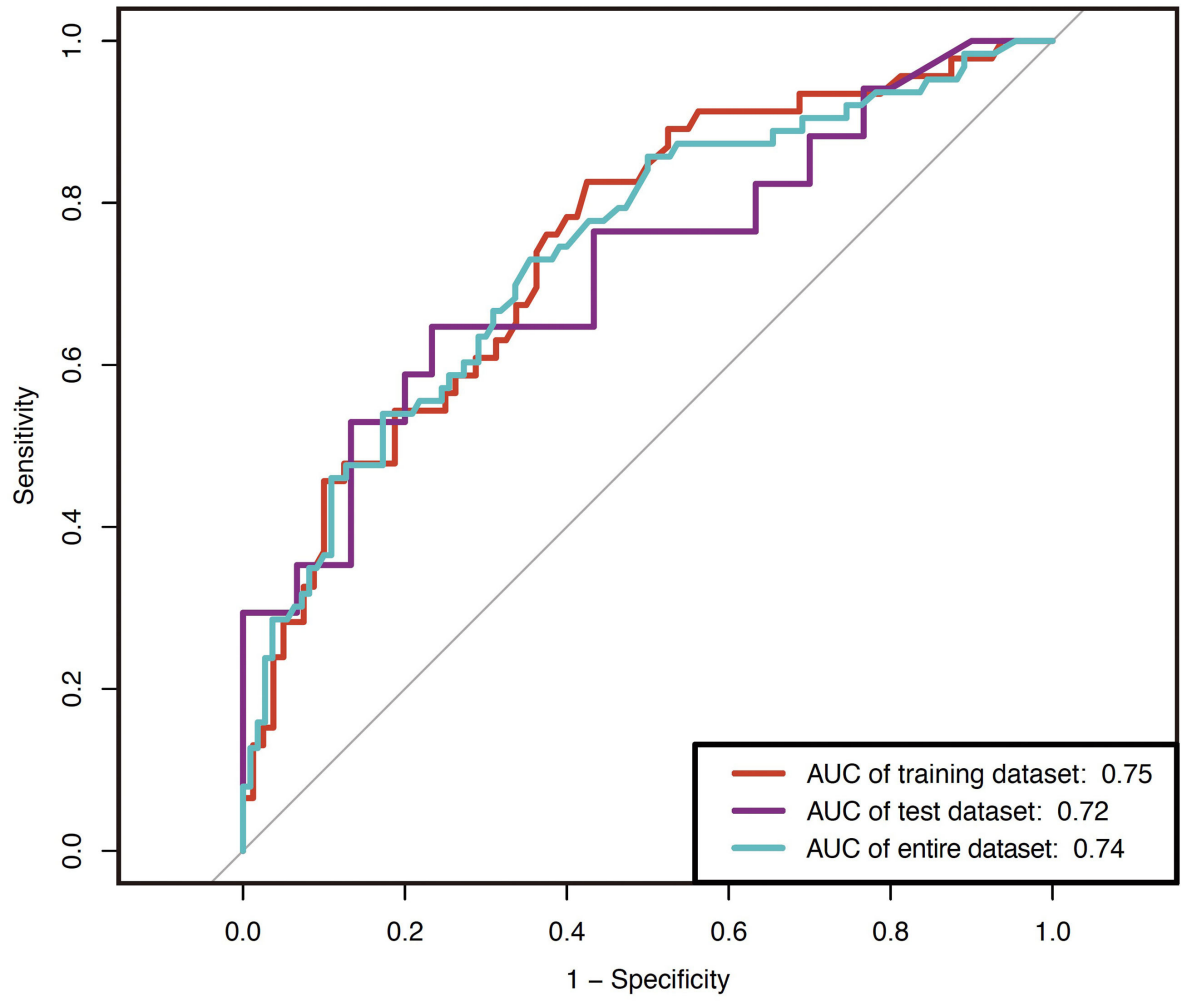
Receiver operating characteristic (ROC) curves of the nomograms in the training data set, validation data set and all data. The nomogram had good discriminative power with an area under the ROC curve of 0.75 (95% CI: 0.66–0.84), 0.72 (95% CI: 0.56–0.88) and 0.74 (95% CI: 0.66–0.82) in the training data set, validation data set and all data, respectively.

**Figure 5:**
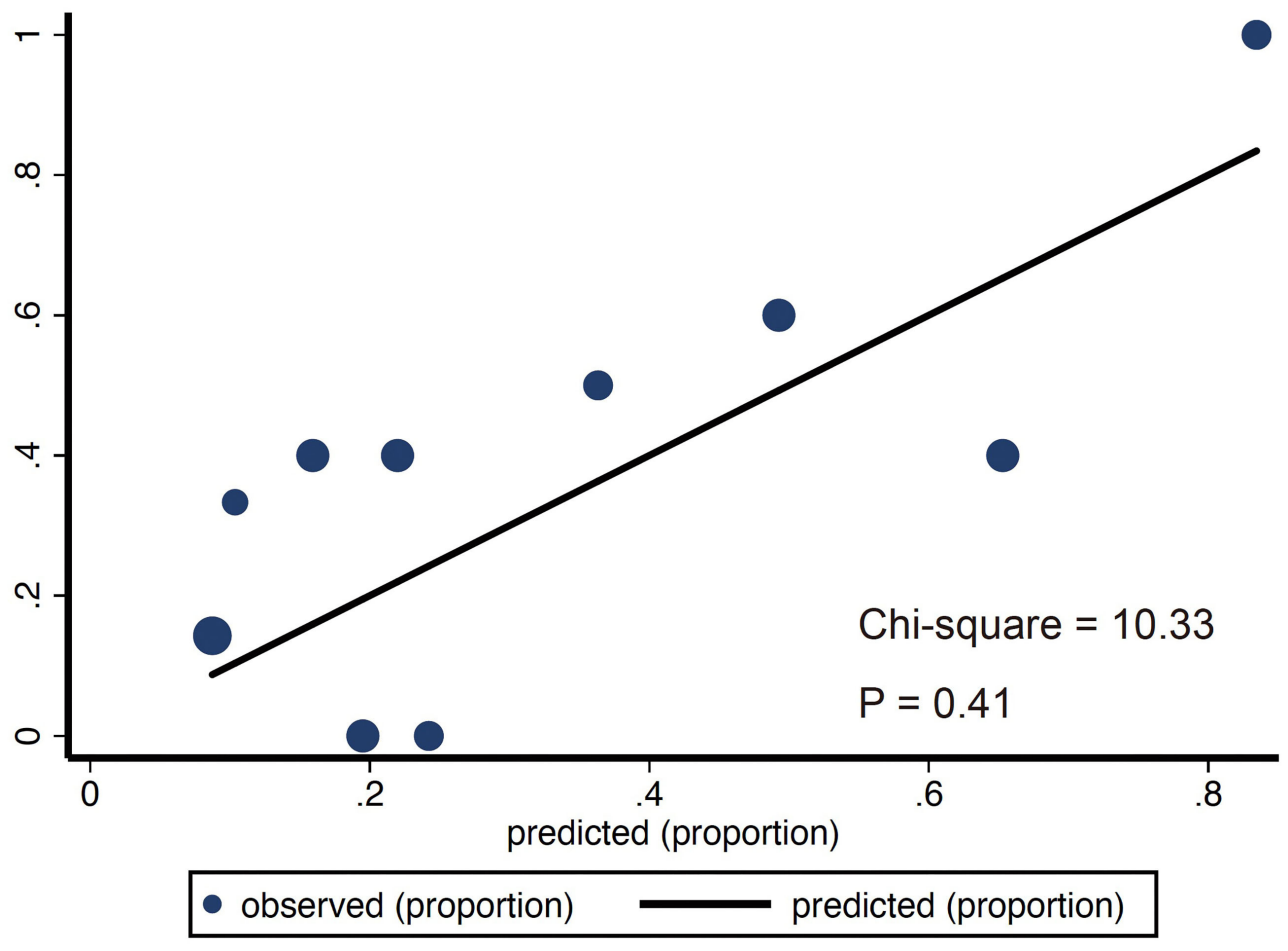
The calibration curve of nomogram for predicting poor prognosis in the validation (*p* = 0.41) dataset. The calibration focused on the accuracy of the absolute risk prediction of the model (i.e., the consistency between the probability of poor prognosis predicted by the model and that actually observed). The y-axis represents the actual rate of poor prognosis. The x-axis represents the predicted probability of poor prognosis. For a nomogram with better calibration, the scatter points should be arranged along a 45° diagonal line. The Hosmer-Lemeshow goodness-of-fit test is often used to compare whether significant differences exist between the prediction probability and the actual occurrence, with *p* > 0.05 indicating no statistically significant difference, and the calibration of the model was good.

We then tested the performance of the model. All evaluations were performed on the test subset data. We assessed the predictive performance of the model using ROC curves. The area under the ROC curve was 0.75 (95% CI: 0.66–0.84) (Figure 4) in the training datasets, indicating good discriminatory of the model. The calibration curve for the nomogram-predicted probability of poor prognosis revealed a favorable agreement between predicted and actual outcomes (Figure 5). The Hosmer-Lemeshow χ2 statistic was 8.42 (*P* = 0.59) in the training dataset.

We next performed validation for the nomogram using the validation set. Here, we applied the resulting model constructed in the training set to calculate the individual probability of poor prognosis. The model showed a good discrimination in the test set (AUC = 0.72, 95% CI: 0.56–0.88) as well as in the entire dataset (AUC = 0.74, 95% CI: 0.66–0.82) (Figure 4). Validation of the calibration curve exhibited good concordance between the nomogram predictions and actual observations [the Hosmer-Lemeshow χ2 statistic was 10.33 (*P* = 0.41)], indicating an excellent fit of the model with the data (Figure 5).

## Discussion

The primary goals of the study were: (1) to develop early predictive model which distinguished poor from good prognosis of patients with AE using a nomogram; and (2) to evaluate nomogram performance using patient-level independent data. We assumed that a set of variables can be recognized through the multivariable regression models and that the simple nomogram based on these predictors had an adequate ability of discrimination and calibration.

In this study, we identified age, viral prodrome, consciousness impairment, memory dysfunction and autonomic dysfunction for predicting poor prognosis of AE at discharge using a multivariable regression model. A nomogram was developed incorporating these predictors presenting both favorable discrimination and adequate calibration.

We found that elderly patients were more likely to have poor prognosis than younger individuals. In previous studies (9, 21), improvement in symptoms of AE after a course of treatment was also shown to be age-related. Multiple organ dysfunction combined with other systemic diseases in elderly people may account for this phenomenon. In addition, delays in time to diagnosis and treatment occurred more frequently in older patients (21). Besides, immunotherapies aim to combat immunosuppression or stimulate adaptive immune response, but elderly people generally respond less well to immunotherapy. Memory disorder was a common sequela of AE (22), and its occurrence at an early state of illness was classified as a predictor in our study. We did not find any other studies that indicated that early decrease in memory is associated with poor prognosis in AE patients. Potentially, this could be attributed to the fact that several previous studies see memory loss as an outcome variable, rather than as an influencing factor contributes to mRS score (10). In the present study, we were surprised to find that viral prodrome was linked with worse outcomes. Two factors may explain these relationships. A case report (23) indicated two patients who were firstly diagnosed with herpes simplex encephalitis but then confirmed as anti-NMDAR encephalitis. This observation demonstrated that prodromal symptoms may contribute to the inflammatory response to viral infection. Besides this, it is possible that prodromal symptoms result from an early immune response (24). Consistent with other studies (25, 26), our research demonstrated that consciousness impairment was also a strong predictor of poor prognosis. A change in the state of consciousness is the most common cause for patients with AE to be admitted to intensive care unit (ICU) (27). In addition, unconscious patients have a worse prognosis probably because of life-threatening complications, such as pneumonia or multiple organ dysfunction syndrome. Autonomic dysfunction is regarded as a disease-specific risk factor and predictor of worse outcome for AE patients, similar to the results of a previous study (15). One hypothesis that can explain such a result is that autonomic dysfunction triggers further intensive care-associated complications such as persistent hypotension or respiratory failure, requiring intubation and mechanical ventilation, even translating into ventilator-associated pneumonia. However, inconsistent with the earlier findings (10), there were no statistically significant correlations between immunotherapy latency and the short-term prognosis, presumably as a result of the small number of cases.

The nomogram, which provides a more individualized prediction, is a statistical tool to graphically illuminate the regression model. A nomogram for assessment of prognosis was developed and integrated using a variety of variables. This enables users to estimate the probability of poor prognosis more accurately. Our nomogram has some notable strengths. This is to our knowledge the first time that a nomogram based on a multicenter dataset has been constructed to effectively predict the prognosis of patients with AE. This nomogram is based only on age and clinical presentation, so other factors such as ancillary examinations are not required to guide model building. This makes our nomogram are easily accessible and simple to integrate into daily clinical practice for patients during the early in-hospital phase. Moreover, the model exhibited good predictive capability in both the training and validation sets.

We recognized that the mRS score at discharge could not represent long-term prognosis, because patients might experience a change in functional status between discharge and long-term outcomes. Assessment at discharge offers a brief snapshot in time allowing clinicians to gauge whether the clinical treatment in hospitalization is effective. Our study helps physicians choose patients who probably receive poor prognosis at discharge, and promote them to give early immunotherapy and intensive monitoring to these patients.

The present study had following limitations. Firstly, the results might be too population-specific because antibody-negative AE were not included. Secondly, in this retrospective study, clinical outcomes were based on physician reports documented in the medical records. Therefore, there was also possible information bias.

## Conclusion

We demonstrated that age, viral prodrome, consciousness impairment, memory dysfunction and autonomic dysfunction were significant predictors for poor prognosis of AE at discharge using a multivariable regression model. The nomogram, deriving from the model, could accurately predict prognosis in patients with AE graphically and provided a personalized outlook. Our study creates a novel model to pick up patients who probably receive poor prognosis and allows physicians to provide a tailored clinical therapeutic regimen for each patient. Precision medicine for AE patients would lead to favorable prognosis and save their medical cost.

## Data Availability Statement

The original contributions generated for the study are included in the article/supplementary material, further inquiries can be directed to the corresponding author/s.

## Author Contributions

QW concepted, designed, and supervised the study. YS, XH, YL, and TW acquired the data. YS analyzed and interpreted the data, provided statistical analysis, had full access to all of the data in the study, and are responsible for the integrity of the data and the accuracy of the data analysis and drafted the manuscript. GR, JR, and QW critically revised the manuscript for important intellectual content. All authors contributed to the article and approved the submitted version.

## Conflict of Interest

The authors declare that the research was conducted in the absence of any commercial or financial relationships that could be construed as a potential conflict of interest.
